# Long-term clinical outcome and satisfaction survey in patients with neurotrophic keratopathy after treatment with cenegermin eye drops or amniotic membrane transplantation

**DOI:** 10.1007/s00417-021-05431-6

**Published:** 2021-10-11

**Authors:** Marta Sacchetti, Chiara Komaiha, Alice Bruscolini, Giuseppe Maria Albanese, Marco Marenco, Rossella Anna Maria Colabelli Gisoldi, Augusto Pocobelli, Alessandro Lambiase

**Affiliations:** 1 Department of Sense Organs, University Sapienza of Rome, Viale del Policlinico, 155, 00161 Rome, Italy; 2 Azienda Ospedaliera San Giovanni Addolorata, Rome, Italy

**Keywords:** Neurotrophic keratitis, Recombinant human nerve growth factor, Cenegermin, Amniotic membrane transplantation, Corneal ulcers

## Abstract

**Purpose:**

Neurotrophic keratopathy (NK) is a degenerative corneal disease caused by damage of trigeminal innervation. The purpose of this study is to evaluate the clinical outcomes and patient-reported satisfaction of treatment with amniotic membrane transplantation (AMT) or cenegermin eye drops in patients with NK.

**Methods:**

Clinical charts of patients with NK treated with AMT (group A) or cenegermin eye drops (group B), with at least 12 months of follow-up, were reviewed for demographics, medical history, corneal healing, and disease recurrence. Patient satisfaction was evaluated by a newly developed questionnaire investigating patient’s appreciation of treatment of NK (2 items) and satisfaction with NK treatment outcomes (5 items).

**Results:**

At the end of treatment, complete corneal healing was observed in 13/15 (86%) patients in group A and in 23/24 (96%) in group B. At 12 months follow-up, 6/13 patients (46%) in group A and 3/23 patients (13%) in group B showed recurrence of NK (*p* = 0.037).

Survival analysis showed that group B remained recurrence free for a significantly longer period of time than the group A (*p* = 0.028). Patients in group B showed a significantly higher satisfaction when compared with patients in group A (total score: 65.7 ± 15.7 vs 47.4 ± 12.8, *p* = 0.003), both in terms of patients’ appreciation of treatment (78.3 ± 15.9 vs 52.2 ± 30, *p* = 0.020) and satisfaction with treatment outcomes (60.7 ± 21 vs 45.4 ± 13.3, *p* = 0.037).

**Conclusions:**

Treatment of NK with cenegermin was associated with long-term maintenance of corneal integrity and a higher degree of patient satisfaction.

**Supplementary Information:**

The online version contains supplementary material available at 10.1007/s00417-021-05431-6.


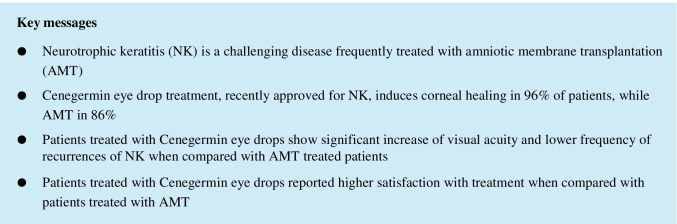


## Introduction

Neurotrophic keratopathy (NK) is a rare, degenerative disease caused by damage of trigeminal innervation and consequent impairment of corneal sensitivity and loss of nerve’s trophic supply to corneal cells [1–3]. Patients with NK develop non-healing corneal epithelial defects, impairment of tear film production and stability, and reduction of blink rate [1]. Clinical presentation of NK ranges from irregular corneal epithelium and/or superficial punctate keratopathy (SPK) (NK stage 1) to epithelial defect (NK at stage 2) and ulcer (NK stage 3), which may progress toward stromal melting and perforation, with loss of visual function [1–3].

The therapeutic approach to NK requires discontinuation of all topical treatments and use of ocular lubricants, which may be associated with application of therapeutic contact lens to help in promoting corneal healing and preventing NK progression [2]. Currently, the only approved medical treatment for NK is cenegermin eye drops (Oxervate®, Dompé Farmaceutici Spa, Milan, Italy), which is a novel drug based on a recombinant human nerve growth factor (rhNGF) [4]. Two different clinical trials showed that cenegermin eye drop treatment was safe and effective in inducing complete cornea healing in more than 70% of patients with NK at stage 2 and 3 [5–8]. Results of these controlled, randomized studies showed that after 12 months of follow-up, more than 87% of patients showing corneal healing after cenegermin eye drops treatment did not experience NK recurrence, suggesting that this treatment is able to induce recovery of corneal nerves and thus to prevent NK progression [6–8]. The introduction of cenegermin eye drops in the ophthalmic pharmacopeia has changed the traditional therapeutic approach to patients with moderate to severe NK, who often required surgical procedures, such as amniotic membrane transplantation (AMT) and/or tarsorrhaphy or conjunctival flap [3, 9, 10]. Small, open label studies showed that these surgical approaches are effective in inducing corneal healing in more than 70% of patients with NK, likewise with low percentages of corneal lesion recurrences during follow-up [11–14]. Since the results of clinical efficacy of both AMT and cenegermin in NK are very similar, it should be of interest to compare AMT with cenegermin in terms of clinical outcomes, recurrence rate, and patient satisfaction. In addition, both treatments have some limitations which may influence the clinicians’ choice, mainly the high costs of cenegermin and the surgical approach of AMT which may also causes temporary impairment of visual function. Therefore, in this study, a patient’s questionnaire has been developed and used to evaluate patients’ satisfaction with cenegermin treatment or AMT. In addition, clinical efficacy and recurrence rate of both treatment with cenegermin eye drops and AMT have been evaluated during 12 months of follow-up.

## Materials and methods

A multicenter, observational study was performed at Department of Sense Organs, University Sapienza of Rome and at San Giovanni Addolorata Hospital of Rome. The clinical charts of all patients with NK treated with cenegermin eye drops or with AMT between January 2017 and January 2020, with at least 12 months of follow-up, were reviewed. The study was performed in accordance with the tenets of the Declaration of Helsinki, and Institutional Review Board/Ethic Committee at the Sapienza University of Rome approval was obtained (code: 5969).

A total of 38 patients met the inclusion criteria: (i) diagnosis of NK at stage 2 (persistent epithelial defect) or stage 3 (corneal ulcer), (ii) previous treatment with AMT (group A) or cenegermin 20mcg/ml eye drops (group B), and (iii) at least 12 months of follow-up after cenegermin eye drops or AMT treatment [3].

Clinical history, including demographical data, NK etiology, and other associated ocular and systemic conditions, as well as previous and current medical and surgical ocular treatments, were recorded at baseline (Table 1). Corneal esthesiometry was assessed at baseline by cotton thread, qualitatively described as normoesthesia, hypoesthesia, or anesthesia [2, 3]. Additional clinical data were also collected including (i) visual acuity assessment with ETDRS chart and (ii) anterior ocular segment slit lamp examination: presence of conjunctival hyperemia, corneal epithelial defect (NK at stage 2), or ulcer (NK at stage 3). These parameters were evaluated at baseline, each week for 2 months and after 12 months of follow-up.

**Table 1:** Demographic and clinical characteristics of the patients with NK included in the study. Group A included patients with NK treated with amniotic membrane transplantation (AMT) and group B included patients with NK treated with cenegermin eye drops

Variable	Group A (AMT *N* = 15)	Group B (cenegermin eye drop *N* = 24)	*p* value
Gender (*N*)			
Male Female	7 8	7 17	0.221
Age (years)			
Mean ± SD	63 ± 15	60 ± 14	0.634
NK etiology (*N*)			
HSV keratitis Post-ocular surgery Dry eye disease Diabetes Post-neurosurgery Ocular caustication Ocular cicatricial pemphigoid Sjogren syndrome	8 1 2 3 1 0 0 0	8 5 3 0 2 2 2 2	-
NK duration (years)			
Mean ± SD	3.7 ± 3.3	4.5 ± 4.9	0.613
Previous treatment for NK (*N*)			
Therapeutic contact lens Cyanoacrylate glue AMT Tectonic lamellar keratoplasty and AMT	2 0 0 1	7 1 1 1	-
Visual acuity—decimal unit			
Mean ± SD	0.1 ± 0.13	0.22 ± 0.22	0.088
NK stage			
2 3	3 12	11 13	0.097
Cornea sensitivity assessed by cotton thread test (*N*)			
Hypoesthesia Anesthesia	5 10	9 15	0.534

Patients in group A were treated with AMT. All cases were affected by NK unresponsive to conventional treatments including use of ocular lubricants and/or therapeutic contact lens. Cryopreserved amniotic membranes were obtained from the Eye Bank of the San Giovanni Addolorata Hospital, Rome (Italy) [15]. AMT was performed mainly under topical and, in selected cases, peribulbar anesthesia [16, 17]. Briefly, in NK stage 2 patients, after removing the loose epithelium surrounding the ulcer with a blunt spatula, amniotic membrane was tailored and placed with the epithelium side up to completely cover the corneal epithelial defect and sutured using nylon 10–0 interrupted *sutures* [17]. Patients with NK stage 3 required additional removal of necrotic debris from the bed of the ulcer by a cellulose swab and filling of the corneal ulcer with tailored amniotic membrane pieces followed by transplantation of an additional piece of amniotic membrane, larger than the ulcer, which was grafted with the epithelium side up and sutured by nylon 10–0 interrupted *sutures* [16]. A therapeutic contact lens was applied, and all patients received preservative-free artificial tears and antibiotic eye drops. Sutures and therapeutic contact lens were removed after complete corneal healing and/or in case of reabsorption and/or loss of the amniotic membrane.

All patients in group B were older than 18 years of age and were diagnosed with NK stage 2 or 3 unresponsive to conventional treatments [3]. They received cenegermin eye drops at 20 μg/ml concentration, 6 times daily for 8 weeks according with treatment protocol [4, 6]. Prophylactic preservative-free antibiotic eye drops were used until the cornea healed. During treatment period, therapeutic contact lens application and additional topical treatments were discontinued with except of anti-glaucomatous eye drops.

All patients in group A and B were allowed to use preservative-free ocular lubricants when needed [3].

Treatments were considered effective if the corneal lesion was completely healed at 2 months. During follow-up period, the development of NK recurrences, defined as the onset of corneal epithelial defect or ulcer, was recorded. All patients with NK recurrence were treated with 14-mm-diameter therapeutic contact lens (CL) application and preservative-free ocular lubricants. Those unresponsive to recurrence treatment with CL application, at the investigator’s discretion, were treated with cenegermin eye drops or with AMT combined with tarsorrhaphy [10]. The clinical outcome of treatments for NK recurrences was also recorded.

### Development of patients’ reported satisfaction (PReS) questionnaire

Patient satisfaction of NK treatment was assessed by using a newly developed questionnaire to be administered to patients with NK by telephone interviews conducted at 12 months of follow-up.

The survey was developed by a multistep process [18]:

1. Items generation. A preliminary list of items which may be included in the survey was developed on the basis of (i) an international literature review to identify existing post-treatment patients’ satisfaction questionnaires and (ii) an interview to a panel of three specialists in treatment of patients with NK which were required to indicate a list of simple questions for administration to the patients.
2. Item reduction. Redundant, difficult to understand, or ambiguous items were qualitatively selected.

The resulting questionnaire included 7 items, aimed at evaluating patients’ satisfaction with treatment of NK with either cenegermin eye drops or AMT (Table 1 in “Supplemental digital content” describes items of the NK treatment satisfaction questionnaire). Specifically, the questionnaire included two items exploring the well-being during the NK treatment period in terms of difficulty to carry out normal daily life activities and the burden of NK treatment for patients and caregivers. The other five items refer to the treatment outcomes in terms of psychosocial well-being (treatment effects on social relationships and quality of life), satisfaction with the clinical outcomes, and, finally, patients’ willingness to repeat the treatment in case of need and to recommend it to someone else affected by NK.

All items were graded on a 5-point Likert scale from 1 to 5 as shown in Table 1 “Supplemental digital content” [19]. The minimum total score possible was 7, representing the worst outcome, and the maximum was 35, representing the best outcome. Summed raw scores were transformed into an equivalent linear Q score on a scale of 0 to 100, with higher scores representing the best outcomes [18].

Cognitive debriefing interviews with 10 patients with NK were performed to verify that the items were clear and easy to understand. The resulting questionnaire was administered to patients with NK included in the study by telephone interviews [18].

### Statistical analysis

Comparison of demographic and clinical parameters between the two groups of treatments was performed by independent sample *t*-test for continuous variables or *χ*2 test for categorical variables. Paired *t*-test was used to compare observations at each follow-up to those at baseline. Additionally, Kaplan–Meier survival estimate was performed to evaluate the recurrence-free time in the 2 treatment groups.

Validity of the questionnaire was evaluated by factorial analysis to examine the underlying association between the 7 items of the questionnaire and to identify potential subscales, the principal component method with Varimax rotation was adopted. Values of factor loading above 0.5 were considered high. Internal consistency of the questionnaire was computed using the Cronbach correlation coefficient, and test–retest reliability was evaluated by using intraclass correlation coefficient (ICC). Measurements with reliability higher than 0.70 were recommended [18]. The statistically significant cut-off value was *p* < 0.05. The statistical analysis was conducted by SPSS software version 22.0 (IBM).

## Results

Clinical characteristics of the patients included in the study are summarized in Table 1. No significant differences in baseline characteristics were observed between the AMT and cenegermin-treated groups, in terms of sex and age as well as duration of NK, NK stage, visual acuity, and corneal sensitivity. According with literature data, the most frequent cause of NK was herpes simplex keratitis, followed by post-surgical trigeminal damage, post-ocular surgery, severe dry eye disease, diabetes, ocular caustication, and ocular cicatricial pemphigoid [2, 6, 14, 20, 21] (Table 1). Associated systemic conditions were reported as well, including diabetes in six patients, high blood pressure in three patients, and atopic diseases in two patients.

At baseline visit, all patients were in treatment with ocular lubricants and prophylactic topical antibiotics. Nine patients also wear therapeutic contact lens. Four patients with glaucoma were also in treatment with topical beta-blockers. Eleven patients with history of recurrent HSV keratitis were also treated with prophylactic systemic antivirals.

**Fig. 1:**
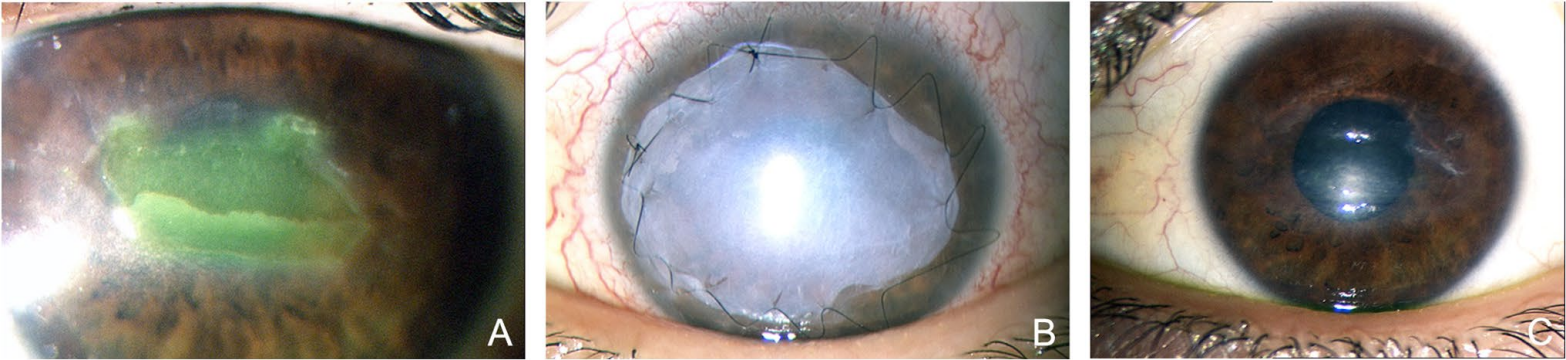
Patient in group A, with NK at stage 3 (**A**), treated with amniotic membrane transplantation (**B**) showed complete corneal healing which was stable over 12 months of follow-up (**C**)

**Fig. 2:**
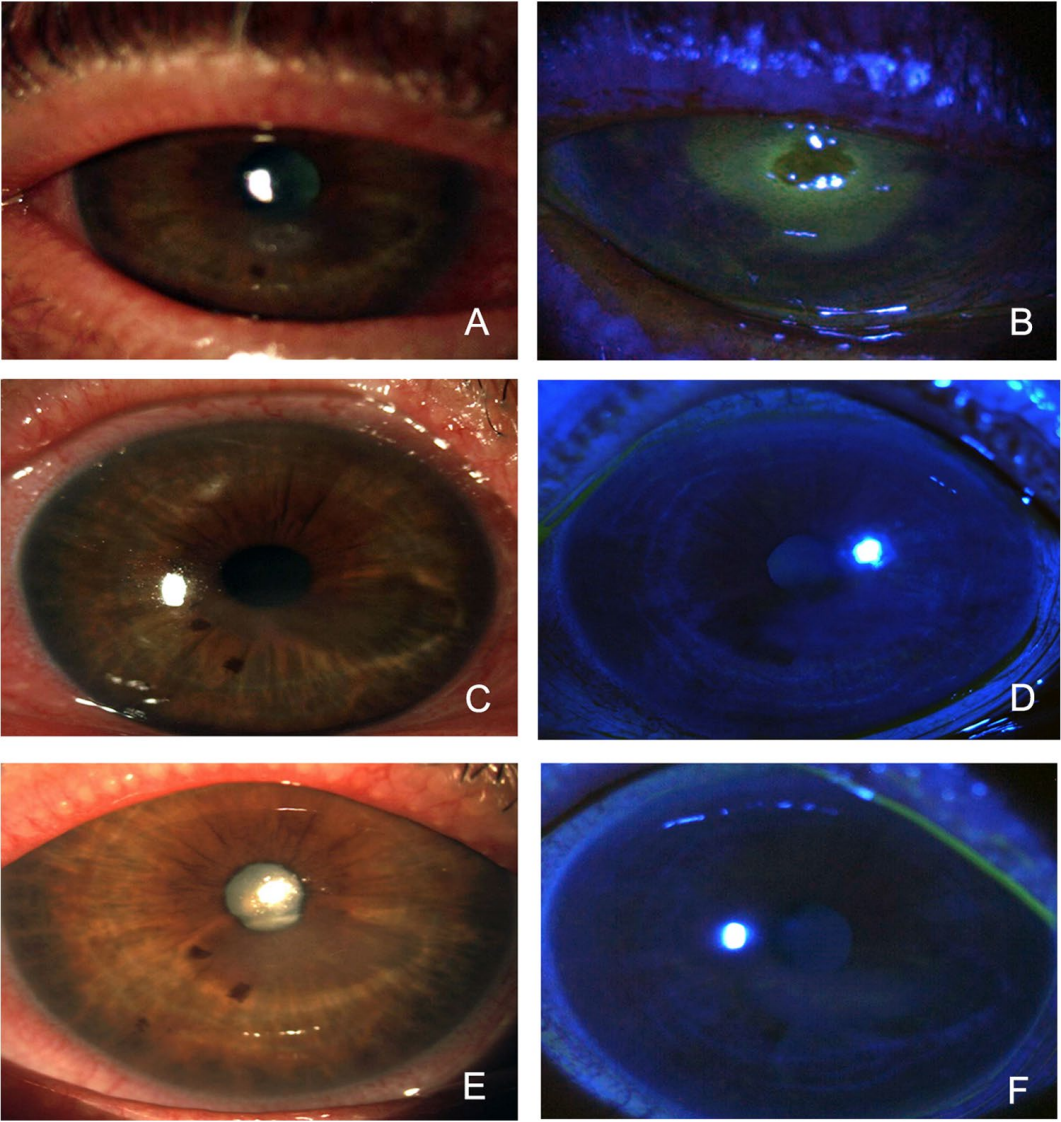
Patient in groups B, with NK at stage 3 (**A**, **B**), showed complete corneal healing after 8 weeks of treatment with cenegermin eye drops (**C**, **D**) and after 12 months of follow-up (**E**, **F**)

At 2 months, 13/15 (86%) patients in group A and 23/24 patients (96%) in group B showed complete corneal healing (Figs. 1, 2). Additionally, the mean time for closure of the corneal lesion was 4.7 ± 3.7 weeks and 4.5 ± 2.2 weeks for group A and group B, respectively. AMT was not effective in 2 patients. These patients showed corneal healing after additional therapeutic contact lens (CL) application in one case and, in the other, after treatment with cenegermin eye drops. In group B, cenegermin eye drop treatment was not effective in 1 patient, in which corneal healing was achieved only after treatment with AMT and partial tarsorrhaphy.

During 12 months of follow-up, 6/13 patients (46%) treated with AMT (group A) and 3/23 patients (13%) treated with cenegermin eye drops (group B) had recurrence of NK (*p* = 0.037).

**Fig. 3:**
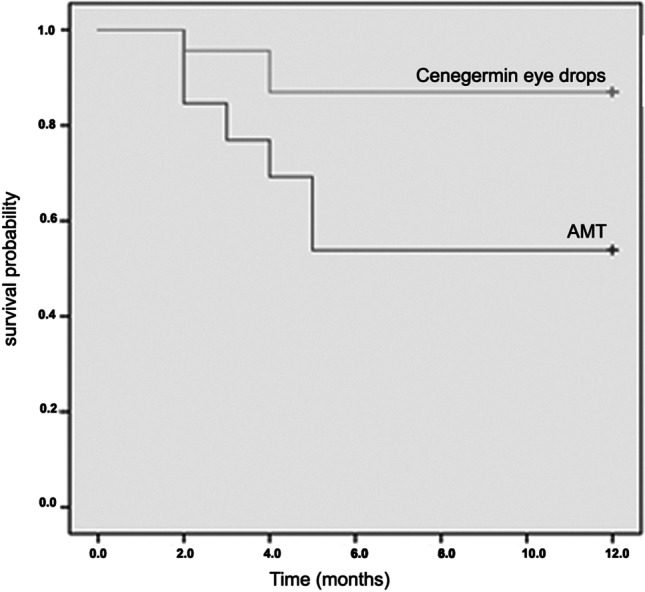
The cenegermin eye drops group had significantly longer recurrence-free periods compared with the AMT group

Survival analysis showed that the cenegermin group remained recurrence free for a significantly longer period of time than the AMT group (*p* = 0.028) (Fig. 3).

All patients with recurrences of NK were initially treated with CL application: six patients from group A and three patients from group B. This was effective in inducing corneal healing in one patient in group A and in all three patients in group B. For the remaining five patients in group A unresponsive to CL application, corneal healing was achieved in two patients treated with cenegermin eye drops alone, two patients treated with AMT combined with tarsorrhaphy, and one patient that underwent tectonic automated lamellar keratoplasty combined with AMT and tarsorrhaphy followed by cenegermin eye drop treatment.

After 12 months of follow-up, BCVA was significantly increased in patients treated with cenegermin eye drops (0.38 ± 0.34 decimal units) when compared with baseline values (*p* = 0.002) but not in patients treated with AMT (0.16 ± 0.24 decimal units). However, the mean change of BCVA between groups after 1 year of follow-up did not show significant difference (mean change BCVA group A 0.05 ± 0.14 vs group B 0.16 ± 0.2, *p* = 0.141).

Patients’ satisfaction was assessed by evaluating responses to the satisfaction survey, which was administered to 26 patients (11 in group A and 15 in group B) by telephone interviews performed by GA and CK. Twelve patients were unable to be reached by telephone after a second phone call and thus did not participate.

Factorial analysis revealed a bidimensional structure of the questionnaire, which explained up to 70.09% of the total variance. The items were therefore allocated into two domains: “satisfaction with treatment outcomes” which includes 5 items exploring patients’ satisfaction with treatment outcomes and “appreciation of treatment,” which includes two items exploring patient’s experience during treatment period (Table 2 Supplemental digital content). Item and scale scores were oriented so that lower scores indicated worse satisfaction. Additionally, linear transformation was performed on questionnaire total scores and on the two domains. The dimensions of the questionnaire showed satisfactory Cronbach alpha values: appreciation of treatment *α* = 0.715 (ICC 0.681) and satisfaction with treatment outcomes *α* = 0.856 (ICC 0.836).

**Fig. 4:**
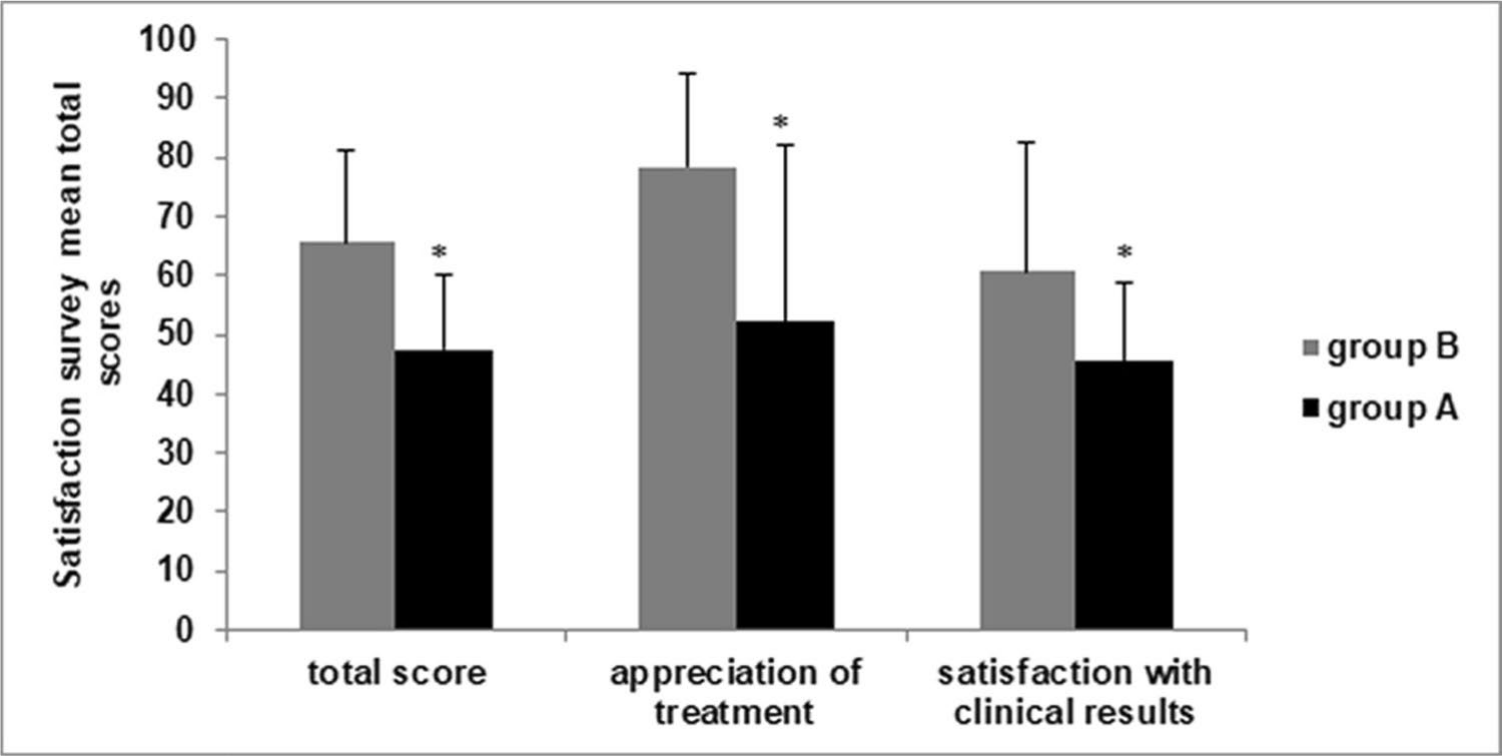
Results of the NK treatment satisfaction questionnaire showed that patients with NK treated with cenegermin eye drops showed higher satisfaction with treatment when compared with patients treated with AMT

Results of the questionnaire showed that patients treated with cenegermin eye drops showed a significantly higher satisfaction when compared with patients treated with AMT (total score: 65.7 ± 15.7 vs 47.4 ± 12.8, *p* = 0.003), both in terms of patients’ appreciation of treatment (78.3 ± 15.9 vs 52.2 ± 30, *p* = 0.020) and satisfaction with treatment outcomes (60.7 ± 21 vs 45.4 ± 13.3, *p* = 0.037) (Fig. 4). Mean scores of the items are shown in Table 3 Supplemental digital content.

## Discussion

This study showed that both treatments, cenegermin eye drops and AMT, were effective in inducing corneal healing in patients with NK. Our data also demonstrated that during 12 months of follow-up, patients treated with cenegermin eye drops showed lower frequency of recurrences and longer periods free of recurrences. Both treatments showed efficacy in the wide spectrum of NK etiology observed in our study population. This eterogenety is in line with the pathogenic mechanisms leading to corneal nerve impairment including surgical, infective, toxic, metabolic, and inflammatory injuries [2, 6, 14, 20, 21].

This is the first study, performed at the same clinical centers, that compared AMT and cenegermin eye drops for the treatment of NK in terms of epithelial healing, long-term clinical outcomes, and patients’ reported satisfaction. Corneal healing rates following treatment with AMT and cenegermin were consistent with previously published data. Specifically, AMT has shown, primarily through relatively small retrospective case series, variable healing rates between 57 and 100% in patients with corneal epithelial defects and ulcers caused by NK [14, 22]. Few studies reported recurrence rate after AMT treatment in NK patients. Specifically, after 12 months, Kruse et al. reported NK recurrence in 2 out 11 patients, and Ivekovic et al. reported complete corneal healing in 11/11 eyes after 12 months of follow-up [16, 23]. Furthermore, evidence from larger randomized controlled trials evaluating cenegermin eye drops have demonstrated healing rates between 69.6 and 74% in patients with moderate to severe NK [6, 7]. Additional follow-up data from these studies showed that 87–96% of patients that received 8 weeks of cenegermin treatment remained recurrence free for up to 12 months [6]. Other studies have reported similar results in terms of epithelial healing and long-term recurrence rate [7, 24–26].

Our results over 12 months of follow-up showed that patients treated with cenegermin eye drops experience significant lower recurrence rate when compared with patients treated with AMT. Specifically, long-term analysis showed that 87% of cenegermin-treated patients remained recurrence free, whereas this was only observed in 53% of AMT-treated patients. Additionally, patients that experienced recurrence after treatment with cenegermin responded to CL application to a much higher degree (100%) than those experiencing recurrence following AMT (16.7%). The higher divergence between the two groups in terms of long-term outcomes and response to recurrence treatment compared to corneal healing immediately post-treatment can potentially be attributed to the difference in the mechanism of each treatment. Specifically, the lower recurrence rate in the cenegermin-treated group suggests that this drug induces corneal recovery by restoring sensory nerve supply, according to previous studies [6, 7, 24, 25, 27–29]. Our results also showed that visual acuity significantly improved after 12 months of follow-up in cenegermin group when compared with baseline values, but not in AMT group. However, comparison of mean changes of visual acuity after 12 months did not show significant difference between groups. This finding may be due to the small number of patients included in this study. In addition to clinical outcomes, patient-reported satisfaction is an important component when selecting treatment for NK, especially when there are visual implications. Surgical procedures, including AMT, while useful, may temporarily impair sight and have negative cosmetic impacts [2, 10]. This study showed that cenegermin was associated with a higher degree of patient satisfaction both in terms of appreciation of therapy and satisfaction with treatment outcomes further reinforcing its clinical utility in the NK treatment paradigm.

The retrospective design and the small sample represent the major limitations of this study. Specifically, the retrospective design does not allow to randomly allocate patients to the two groups of treatment with potential selection bias and/or allocation bias. Although at baseline clinical and demographical characteristics of study populations, including NK stage and duration, did not show statistically significant difference between groups, the higher percentage of NK stage 3 in group B (80%) than in group A (54%), and the more difficult management of the recurrences during follow-up in patients treated with AMT, may suggest that more difficult cases were treated with AMT. It is worth to note that currently no standardized treatment guidelines are available, and treatment choice is mostly the result of physicians’ considerations as well as of patient preference. By this point of view, our results may provide physicians with some information on long-term efficacy of these two therapeutic approaches in order to help treatment choice and potentially improve the management of NK. Prospective, larger, randomized clinical trials with standardization of procedures and evaluations, including quantitative assessment of corneal sensitivity, are required to confirm our observation and provide data useful to establish the treatment decision tree for this challenging condition.

Our results, both from the perspective of clinical outcomes and from the patients’ reported satisfaction with treatment, suggest that the use of cenegermin should be considered a first-line therapy for moderate to severe NK.

However, the high costs of cenegermin, which is biologic drug that requires cold chain storage and delivery, may represent a limitation for its broad clinical use. Currently, cenegermin is widely available in the USA where it is reimbursed by most insurance companies. It is also available throughout Europe as it was approved at a central level by EMA, and recently it was also approved by the Swiss health authority [4]. However, negotiations on price reimbursement are still ongoing at local level in different European countries and in Switzerland. As consequence, cenegermin, even if it is the only approved drug for NK treatment, shows a limited clinical use, and several surgical approaches, such as tarsorrhaphy, sutured and sutureless AMT, or conjunctival flap as well as medical treatments such as blood-derived eye drops including autologous serum, umbilical serum, and PRP, still remain the only treatment choices for NK patients in many parts of the world [2, 14, 22]. Currently, the use of blood-derived eye drops is still limited by the difficulty to establish an optimal concentration and dose regimen as well as by risk of contamination and limited accessibility [13, 29, 30].

In conclusion, this study confirms that AMT and cenegermin are equally effective in inducing corneal healing in both stage 2 and 3 NK patients and that cenegermin treatment is associated with minor frequency of recurrences and higher patient satisfaction.

## Supplementary Information

Below is the link to the electronic supplementary material.

Supplementary file1 (DOCX 47 KB)

## Data Availability

Data are available by the corresponding author at request.
