# Supplementary material for: Long-term clinical outcome and satisfaction survey in patients with neurotrophic keratopathy after treatment with cenegermin eye drops or amniotic membrane transplantation
Source: Graefes Arch Clin Exp Ophthalmol. 2021 Oct 11;260(3):917–25. doi: 10.1007/s00417-021-05431-6 (PMC8850233; doi:10.1007/s00417-021-05431-6)
Supplement: Supplementary file 1 — Supplementary file1 (DOCX 47 KB) [file 417_2021_5431_MOESM1_ESM.docx]

**Table 1 Supplemental digital content. List of the 7 items and their rating scale (form 1 to 5) included in the final version of the NK treatment satisfaction questionnaire used for the phone survey in this study (translated from Italian).**

|  | **Responses** |
| --- | --- |
| ***Treatment performed for NK:***   1. Cenegermin eye drops 2. Amniotic membrane transplantation | Yes No date__/__/____  Yes No date__/__/____ |
| **Items** | **Scores** |
| 1. During the treatment period, did you experience difficulty in carrying out the normal daily life activities (e.g. working, taking care of your family, taking care of the house, practicing your hobbies, etc.)? | **Never Rarely Sometimes Often Always**  5 4 3 2 1 |
| 2. How challenging was the treatment of NK for you and your caregivers? | **Not at all Not much Partially Much Very much**  5 4 3 2 1 |
| 3. After treatment, did your social relationships improve? | **Not at all Not much Partially Much Very much**  1 2 3 4 5 |
| 4. After treatment, did your quality of life improve when compared with the period before treatment? | **Not at all Not much Partially Much Very much**  1 2 3 4 5 |
| 5. Are you satisfied with the treatment you have received? Did the treatment work? | **Not at all Not much Partially Much Very much**  1 2 3 4 5 |
| 6. In case of need, how willing would you be to repeat the treatment? | **Not at all Not much Partially Much Very much**  1 2 3 4 5 |
| 7. Would you recommend someone with neurotrophic keratopathy to undergone the same treatment as you? | **Not at all Not much Partially Much Very much**  1 2 3 4 5 |

**Table 2 Supplemental digital content. Factorial analysis of the NK treatment satisfaction questionnaire items, using Varimax rotation method, allowed allocation of the 7 items into two domains: factor I “satisfaction with treatment outcomes” (items 3,4,5,6 and 7) and factor II “appreciation of treatment” (items 1 and 2)**.

| **Items** | **Factor I: “Satisfaction with treatment outcomes”**  **Loading*** | **Factor II: “Appreciation of treatment “**  **Loading*** |
| --- | --- | --- |
| 1. During the treatment period, did you experience difficulty in carrying out the normal daily life activities (eg working, taking care of your family, taking care of the house, practicing your hobbies, etc.)? | 0.101 | **0.847*** |
| 2. How challenging was the treatment of NK for you and your caregivers? | 0.003 | **0.883*** |
| 3. After treatment, did your social relationships improve? | **0.626*** | 0.071 |
| 4. After treatment, did your quality of life improved when compared with the period before treatment? | **0.863*** | -0.134 |
| 5. Are you satisfied with the treatment you have received? Did the treatment work? | **0.790*** | -0.073 |
| 6. In case of need, how willing would you be to repeat the treatment? | **0.794*** | 0.393 |
| 7. Would you recommend someone with neurotrophic keratopathy to undergone the same treatment as you? | **0.873*** | 0.246 |
| **% variance**  **Total variance:** **70.09** | 45.21 | 24.87 |

*Items were considered related to each Factor if loaded higher than 0.5.

**Table 3 Supplemental digital content. Mean response scores (graded from 1 to 5) to the 7 items of the NK treatment satisfaction survey administered to 11 patients treated with amniotic membrane tranplatation (group A) and 15 patients treated with Cenegermin eye drops (group B).**

| Item | Group A (N=11) | Group B (N=15) | P value |
| --- | --- | --- | --- |
| 1. During the treatment period, did you experience difficulty in carrying out the normal daily life activities (eg working, taking care of your family, taking care of the house, practicing your hobbies, etc.)? | 3.1±1.6 | 4.5±0.8 | 0.027* |
| 2. How challenging was the treatment of NK for you and your caregivers? | 3±1 | 3.7±0.7 | 0.048* |
| 3.After treatment, did your social relationships improve? | 1.5±0.7 | 2.3±1.4 | 0.064 |
| 4.After treatment, did your quality of life improved when compared with the period before treatment? | 2.5±0.9 | 3±1.4 | 0.264 |
| 5. Are you satisfied with the treatment you have received? Did the treatment work? | 3.3±1 | 3.4±1.1 | 0.813 |
| 6. In case of need, how willing would you be to repeat the treatment? | 3.3±0.5 | 4.1±0.8 | 0.008* |
| 7.Would you recommend someone with neurotrophic keratopathy to undergone the same treatment as you? | 3.2±0.5 | 4.1±0.7 | 0.001* |

*statistically significant
